# Clinical Course and Surgical Outcomes of Patients with Tetralogy of Fallot with Absent Pulmonary Valve Syndrome: A Single-Center Retrospective Study

**DOI:** 10.3390/jcm15176586

**Published:** 2026-08-26

**Authors:** Şerif Şerifoğlu, Fatma Sevinç Şengül, Mustafa Nalbant, Buse Günyel, Yunus Emre Sarı, Mehmet Balcı, Pelin Ayyıldız, Aysel Türkvatan Cansever, Okan Yıldız, İsmihan Selen Onan, Sertaç Haydin, Alper Güzeltaş

**Affiliations:** 1 Department of Pediatric Cardiology, Istanbul Mehmet Akif Ersoy Thoracic and Cardiovascular Surgery Training and Research Hospital, University of Health Sciences, Istanbul 34303, Turkey; 2 Department of Pediatric Cardiology, Manisa City Hospital, Manisa 45040, Turkey; 3 Department of Pediatric Cardiology, Hatay Training and Research Hospital, Hatay 31027, Turkey; 4 Department of Radiology, Mehmet Akif Ersoy Thoracic and Cardiovascular Surgery Training and Research Hospital, University of Health Sciences, Istanbul 34303, Turkey; 5 Department of Pediatric Heart Surgery, Istanbul Mehmet Akif Ersoy Thoracic and Cardiovascular Surgery Training and Research Hospital, University of Health Sciences, Istanbul 34303, Turkey

**Keywords:** tetralogy of Fallot, pulmonary valve, heart defects, congenital, pulmonary artery, airway obstruction, respiration, artificial, cardiac surgical procedures, reoperation

## Abstract

**Background:** Tetralogy of Fallot with absent pulmonary valve syndrome (TOF-APVS) is a rare variant of tetralogy of Fallot marked by aneurysmal pulmonary artery dilatation and variable tracheobronchial compression. The identification of clinical indicators that predict perioperative risk in this patient population remains challenging. We aimed to review the surgical outcomes and reintervention requirements of patients with TOF-APVS over a 15-year single-center experience, with particular focus on two indicators of disease severity: preoperative mechanical ventilation (MV) and computed tomography (CT)-detected airway compression. **Methods:** We retrospectively reviewed 27 patients with TOF-APVS who underwent surgical repair at a single center between January 2010 and January 2025. Airway compression was assessed by CT in 20 patients. The primary outcome was the need for any reintervention during follow-up. Secondary outcomes comprised early and overall mortality and postoperative duration of intubation, ICU stay, and hospital stay. Subgroup comparisons were performed using the Fisher exact test and the Mann–Whitney U test. **Results:** The median age at surgery was 8 months (IQR, 4.5–16). Preoperative MV was required in 5 patients (18.5%), and CT revealed airway compression in 13 of 20 patients (65.0%). Early postoperative mortality was 7.4%, and overall mortality during follow-up was 18.5%. At least one reintervention was required in 9 patients (33.3%). Preoperative MV was significantly associated with early mortality (40.0% vs. 0%; *p* = 0.028) and with prolonged ICU stay (median 15 vs. 4 days; *p* = 0.008). CT-detected airway compression was not significantly associated with either mortality or reintervention. **Conclusions:** In this 15-year single-center experience with 27 patients, preoperative mechanical ventilation was associated with markedly higher early postoperative mortality and prolonged ICU stay, and may thus serve as a practical clinical indicator of disease severity in TOF-APVS. CT-detected airway compression was a frequent and often severe finding, but its association with early mortality and reintervention could not be statistically confirmed, likely reflecting the selective use of CT and small subgroup size. These findings support the integration of preoperative ventilation status into perioperative risk assessment and highlight the need for larger, preferably multicenter, studies with standardized airway assessment to clarify the prognostic role of anatomical airway compression.

## 1. Introduction

**Figure 1:**
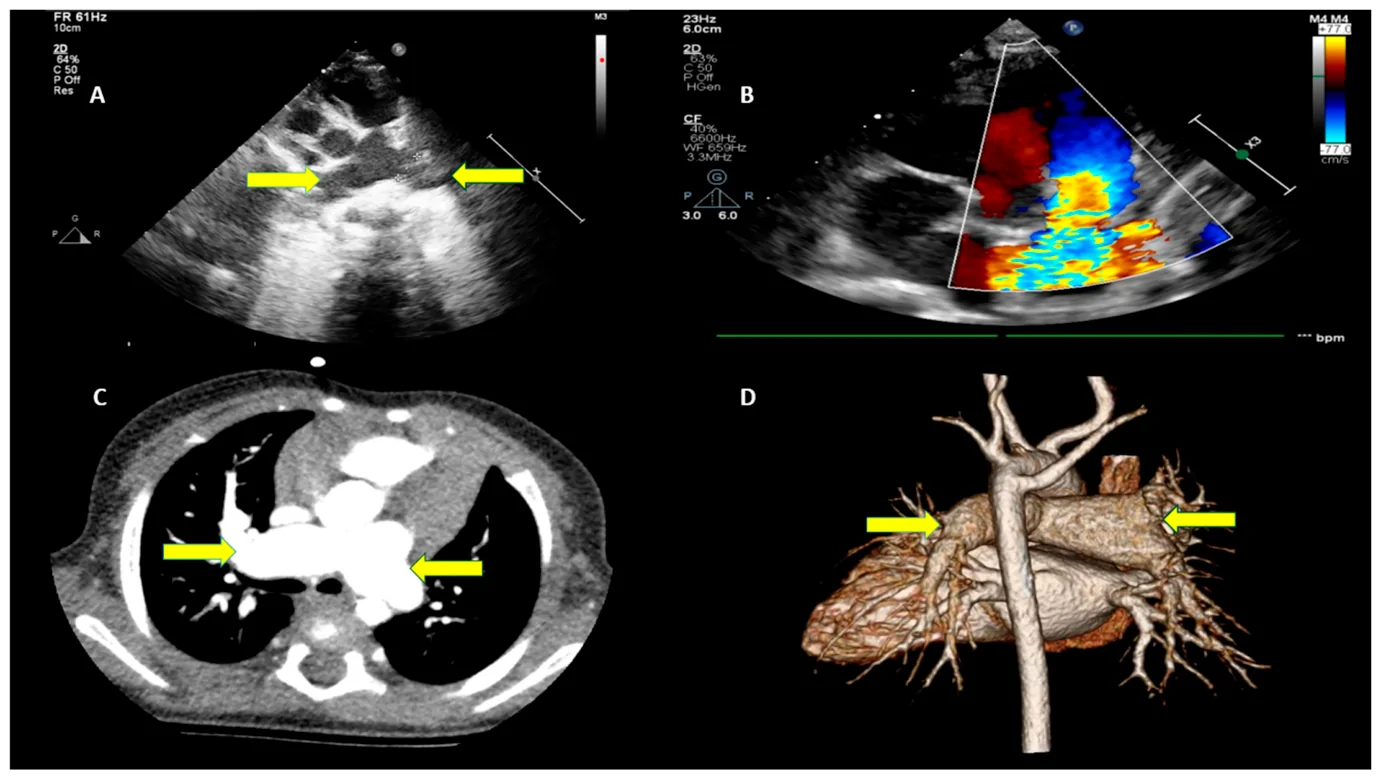
Multimodality imaging findings in a patient with tetralogy of Fallot with absent pulmonary valve syndrome (TOF-APVS), demonstrating aneurysmal dilatation of the main and branch pulmonary arteries. (**A**) Transthoracic echocardiography, two-dimensional parasternal short-axis view, showing markedly dilated main and branch pulmonary arteries (arrows). (**B**) Transthoracic echocardiography, parasternal short-axis view with color Doppler interrogation, demonstrating turbulent flow within the dilated pulmonary arteries consistent with free pulmonary regurgitation. (**C**) Contrast-enhanced computed tomography (CT), axial view at the level of the pulmonary bifurcation, showing aneurysmal dilatation of the main pulmonary artery and both branch pulmonary arteries (arrows). (**D**) Three-dimensional CT reconstruction (volume-rendered image) illustrating the aneurysmal dilatation of the main pulmonary artery and its branches (arrows) and their spatial relationship to the surrounding mediastinal structures.

Congenital heart disease (CHD) affects approximately 8–10 per 1000 live births globally and represents one of the most common groups of congenital malformations [1]. Tetralogy of Fallot (TOF) accounts for approximately 3–7% of all CHD, making it the most common cyanotic congenital cardiac defect [2]. Tetralogy of Fallot with absent pulmonary valve syndrome (TOF-APVS) is an uncommon variant, comprising approximately 3–6% of all cases of tetralogy of Fallot [3]. In Turkey, a nationwide surveillance study of critical congenital heart disease (CCHD) reported an overall CCHD prevalence of 27.8 per 10,000 live births, with TOF accounting for 17.4% of cases and representing the most frequent critical CHD subtype [4]. Tetralogy of Fallot with absent pulmonary valve syndrome (TOF-APVS) presents with a malalignment-type ventricular septal defect (VSD), varying degrees of pulmonary annular stenosis, rudimentary or entirely absent pulmonary valve tissue, and the resulting free pulmonary regurgitation. Its hallmark finding is aneurysmal dilatation of the main pulmonary artery (MPA) and its branches, which may compress the tracheobronchial tree and lead to respiratory morbidity [5] (Figure 1). TOF-APVS represents a rare congenital cardiac anomaly with a broad clinical spectrum [6]. Airway-related morbidity and mortality stand among the main features that distinguish TOF-APVS from other variants of the TOF spectrum [7]. While some patients remain asymptomatic and receive their diagnosis incidentally during adolescence, others develop severe respiratory failure soon after birth [8].

In neonates and young infants with severe respiratory compromise or signs of cardiac decompensation, preoperative mechanical ventilation (MV) and early surgical repair may become necessary. Surgical management often involves pulmonary artery (PA) plasty of varying extent—from moderate PA reduction to extensive intrahilar plication—determined by the degree of PA dilatation and the severity of airway involvement. Postoperatively, the clinical course in this subgroup can be complicated by respiratory complications, prolonged ventilator dependency, tracheobronchomalacia, and recurrent respiratory tract infections secondary to air trapping [9,10].

Patients with minimal or no airway symptoms, on the other hand, present with a clinical picture that resembles classic TOF, and postponing surgical repair to 6–12 months of age has been linked to more favorable postoperative and long-term outcomes in several series [8,11,12,13]. Nevertheless, in this subgroup with stable respiratory status, the necessity of PA reduction and the optimal surgical approach remain a matter of debate [10].

Survival of patients with TOF-APVS has improved considerably over the past decades; postoperative mortality in contemporary series is now commonly reported around 10–20% [3,9]. A substantial proportion of deaths occur in neonates and infants with severe respiratory distress, and preoperative ventilator dependency has emerged as an important risk factor for adverse outcomes and increased mortality [14,15]. Despite these improvements in survival, reintervention rates remain considerable, and many patients require multiple reinterventions targeting the right ventricular outflow tract (RVOT) and/or the pulmonary arteries during follow-up [13,15]. A clearer definition of clinical severity indicators and of the factors that shape the course of follow-up is therefore warranted.

Although preoperative mechanical ventilation and computed tomography (CT)-detected airway compression are increasingly recognized as clinically important features in TOF-APVS, previous studies have generally examined these two parameters in isolation or as descriptive findings within broader surgical cohorts, rather than as interrelated indicators of disease severity. Comprehensive single-center analyses that concurrently evaluate both indicators, and that report airway compression not only as present or absent but also with detailed anatomical characterization (location and severity), remain scarce in the literature. This absence of integrated risk stratification data limits the ability of clinicians to identify perioperative high-risk subgroups in this rare and heterogeneous population.

In view of this gap, the aim of the present study was to review our 15-year single-center experience with TOF-APVS and to concurrently evaluate two indicators of disease severity—preoperative mechanical ventilation requirement and CT-detected airway compression, characterized by location and severity—with respect to their association with early and late surgical outcomes, mortality, and reintervention requirements. As one of the largest national referral centers for congenital cardiac surgery, our cohort offers a contemporary contribution to the limited body of evidence on this rare congenital anomaly.

## 2. Materials and Methods

### 2.1. Study Design and Patient Selection

We conducted a single-center, retrospective, observational study at a tertiary cardiac referral center. Eligible patients had a confirmed diagnosis of tetralogy of Fallot with absent pulmonary valve syndrome and underwent surgical repair at our institution between January 2010 and January 2025. During the 15-year study period, 27 consecutive patients with a confirmed diagnosis of TOF-APVS underwent surgical repair at our institution. The inclusion criteria comprised: (i) a confirmed diagnosis of TOF-APVS based on echocardiographic and, where available, CT or catheterization findings, and (ii) surgical repair performed at our institution during the study period. Patients with incomplete perioperative or follow-up data precluding meaningful analysis would have been excluded, but no such cases were identified; all 27 patients therefore comprised the final study cohort. Diagnosis relied primarily on transthoracic echocardiography (TTE) and, when available, was corroborated by operative findings, CT, or catheterization data. All diagnoses were established or confirmed by pediatric cardiologists at our institution. For the purposes of this study, TOF-APVS was defined by the characteristic combination of a malalignment-type VSD, RVOT obstruction of variable severity, rudimentary or absent pulmonary valve tissue with free pulmonary regurgitation, and aneurysmal dilatation of the main and/or branch pulmonary arteries, in accordance with previously published definitions [5,7,16]. CT and catheterization were not mandatory for inclusion, as they were performed selectively based on clinical need. No patient who met the inclusion criteria was excluded due to missing core clinical, operative, or follow-up data.

### 2.2. Ethical Approval, Consent, and Data Confidentiality

This investigation was conducted in accordance with the ethical standards laid down in the Declaration of Helsinki, and institutional ethics approval was granted by the Ethics Committee of the Istanbul Mehmet Akif Ersoy Thoracic and Cardiovascular Surgery Training and Research Hospital (Istanbul, Turkey) (decision no.: 2025.06-54; date: 30 June 2025). All analyses were performed on de-identified datasets; personal patient information remained confidential throughout the study and was accessed exclusively for research-related purposes. Because of the retrospective study design and the exclusive use of anonymized records, the ethics committee waived the need for individual informed consent.

### 2.3. Clinical Data and Definitions

Demographic and clinical characteristics—age at presentation, body weight, and preoperative clinical findings (cyanosis, respiratory distress, history of recurrent infections, and need for intensive care)—were recorded. Preoperative mechanical ventilation (MV) was defined as the need for invasive mechanical ventilatory support via endotracheal intubation prior to surgical repair. In-hospital complications were retrieved from medical records.

### 2.4. Echocardiographic Evaluation

TTE served as the principal diagnostic modality in all patients. Echocardiographic assessment addressed the presence and location of the VSD, the degree of aortic override, the anatomy and degree of obstruction of the RVOT, the severity of pulmonary stenosis (PS) and pulmonary regurgitation (PR), the pulmonary valve annulus diameter, and the diameters of the main, right, and left pulmonary arteries (MPA, RPA, and LPA). Z-scores for the pulmonary annulus and pulmonary arteries were derived from the reference values reported by Pettersen et al. [17].

### 2.5. CT and Catheter-Based Imaging—Indications

CT and cardiac catheterization were not part of the routine workup. CT was reserved for selected patients with marked respiratory symptoms and/or suspected airway compression, to enable a detailed evaluation of tracheobronchial compression and pulmonary artery anatomy. Cardiac catheterization was carried out when clinically indicated, either for hemodynamic assessment or as part of an interventional treatment plan.

### 2.6. CT Measurements and Assessment of Airway Compression

In patients who underwent CT, the diameters of the main and branch pulmonary arteries were documented. RPA and LPA measurements were taken in the pre-hilar segment, before branching. Airway compression was evaluated on CT images. Luminal narrowing of the trachea and/or main bronchi consistent with extrinsic compression was defined as “airway compression” and coded as a binary variable (present/absent). The site of compression was classified as involving the trachea, the right main bronchus, the left main bronchus, or combined sites. The degree of luminal narrowing on CT was calculated as a percentage relative to the adjacent normal segment and graded as mild (<25%), moderate (25–75%), or severe (>75%). For subgroup comparisons, the compression variable was used in its dichotomous form (present/absent); details of location and severity were reported descriptively. CT findings were extracted from original radiology reports and operative planning records.

### 2.7. Associated Anomalies and Genetic Evaluation

Associated cardiac and extracardiac anomalies (e.g., aortic arch anomalies, coronary artery anomalies, and genetic diagnoses) were retrieved from echocardiographic findings, CT/catheterization reports, and operative notes. The presence of major aortopulmonary collateral arteries (MAPCAs) was reviewed from the records and reported accordingly.

### 2.8. Surgical Technique and Postoperative Follow-Up

Details of the surgical repair were obtained from operative notes. The type of RVOT reconstruction (e.g., transannular patch or conduit reconstruction), pulmonary valve strategy (valve replacement and valve type), and pulmonary artery procedures (PA reduction/reconstruction; plication/reduction arterioplasty) were documented. Postoperative duration of intubation (days), length of intensive care unit (ICU) stay (days), length of hospital stay (days), and complications were assessed. Early mortality was defined as death occurring within 30 days after surgery or before hospital discharge. Deaths that occurred during follow-up were also recorded and included in the overall mortality.

### 2.9. Catheterization, Reintervention, and Outcomes

Catheter-based reinterventions performed during follow-up were grouped as “catheter reintervention”; percutaneous pulmonary valve implantation (PPVI), balloon angioplasty, and balloon angioplasty with stent placement were each recorded separately.

The primary outcome was the need for any reintervention during follow-up—a composite endpoint comprising early reoperation, late reoperation, and/or catheter reintervention. Secondary outcomes included early mortality (≤30 days or before discharge), overall mortality, performance of PPVI, and postoperative duration of intubation, ICU stay, and hospital stay. The individual components of the composite primary outcome (early reoperation, late reoperation, and catheter reintervention) and the number of catheter reinterventions were also reported separately.

### 2.10. Data Collection and Missing Data

Follow-up data were obtained from routine outpatient cardiology visits and institutional medical records. Vital status was ascertained for all 27 patients (100%); mortality events during follow-up were confirmed through institutional death records. Among the 22 surviving patients, long-term outpatient clinical follow-up beyond the first 6 months was available for 21 patients; 1 patient (3.7% of the total cohort) was lost to long-term clinical follow-up after being discharged in stable condition, with the last documented outpatient visit occurring 4 months after surgery.

### 2.11. Statistical Analysis

All data analyses were completed on IBM SPSS Statistics, version 26.0 (IBM Corp., Armonk, NY, USA). For continuous variables, we reported median with interquartile range (IQR) or mean with standard deviation, according to the observed distribution pattern; categorical data were summarized as absolute frequencies with percentages. When expected frequencies were small, categorical variables were compared through the Fisher exact test. Between-group differences in continuous variables were tested by the Mann–Whitney U procedure. For the main between-group comparisons of proportions, exact 95% confidence intervals (CI) were calculated using the Clopper–Pearson method. Freedom-from-reintervention was estimated using the Kaplan–Meier method, with deaths and loss to follow-up treated as censoring events. A two-tailed *p* threshold below 0.05 defined statistical significance. Because the number of patients was limited, subgroup analyses were treated as exploratory and were performed primarily to detect clinically meaningful patterns; on this basis, no adjustment for multiple testing was performed, and the corresponding *p* values were interpreted cautiously. A formal a priori power calculation was not performed, given the rarity of TOF-APVS and the observational retrospective design; accordingly, results are interpreted with attention to CI width rather than solely to *p* values.

## 3. Results

### 3.1. Overall Cohort Characteristics (N = 27)

A total of 27 patients with tetralogy of Fallot–absent pulmonary valve syndrome (TOF-APVS) were included in the study. Seventeen patients (63.0%) were male. Preoperative mechanical ventilation was required in 5 patients (18.5%). CT was performed in 20 patients (74.1%), and cardiac catheter angiography was performed in 13 patients (48.1%). The median age at diagnosis was 3 months (IQR, 1–8 months; range, 0–360 months), and the median age at surgery was 8 months (IQR, 4.5–16 months; range, 0–432 months). Both maximum values reflect the same patient, in whom surgical repair was delayed for several years after the initial diagnosis due to loss to follow-up, and who subsequently underwent surgical repair after re-presentation at our institution. The median body weight at presentation was 4.8 kg (IQR, 4.0–6.0 kg; range, 2–55 kg) (Table 1).

The median pulmonary annulus diameter was 7 mm (IQR, 6–8 mm; range, 4–17 mm), with a median Z-score of −2.24 (IQR, −3 to 0; range, −8 to 4). The median main pulmonary artery (MPA) diameter was 16 mm (IQR, 12–21 mm; range, 6–44 mm), with a median Z-score of 2.9 (IQR, 2–5; range, −4 to 7). The median right pulmonary artery (RPA) diameter was 14.5 mm (IQR, 10–20 mm; range, 6–32 mm), with a median Z-score of 5.58 (IQR, 4–7; range, 1.4–13). The median left pulmonary artery (LPA) diameter was 13 mm (IQR, 9–17 mm; range, 5–30 mm), with a median Z-score of 5.5 (IQR, 4–7; range, 1–10) (Table 2) (Figure 1).

A VSD was present in all patients (n = 27, 100%). Coexisting atrial septal defect (ASD) was identified in 17 patients (63.0%) and patent ductus arteriosus (PDA) in 3 patients (11.1%). A left-sided aortic arch was present in 18 patients (66.7%) and a right-sided aortic arch in 9 patients (33.3%). Right ventricular dilatation was identified in 15 patients (55.6%), whereas right ventricular dysfunction was not observed in any patient. Tricuspid regurgitation was mild in 5 patients (18.5%); the remaining 22 patients (81.5%) had no tricuspid regurgitation (Table 2). Major aortopulmonary collateral arteries (MAPCAs) were identified in 6 patients (22.2%) and were managed by intraoperative ligation in all cases.

### 3.2. CT Subgroup Analysis (N = 20): Findings Related to Airway Compression

**Figure 2:**
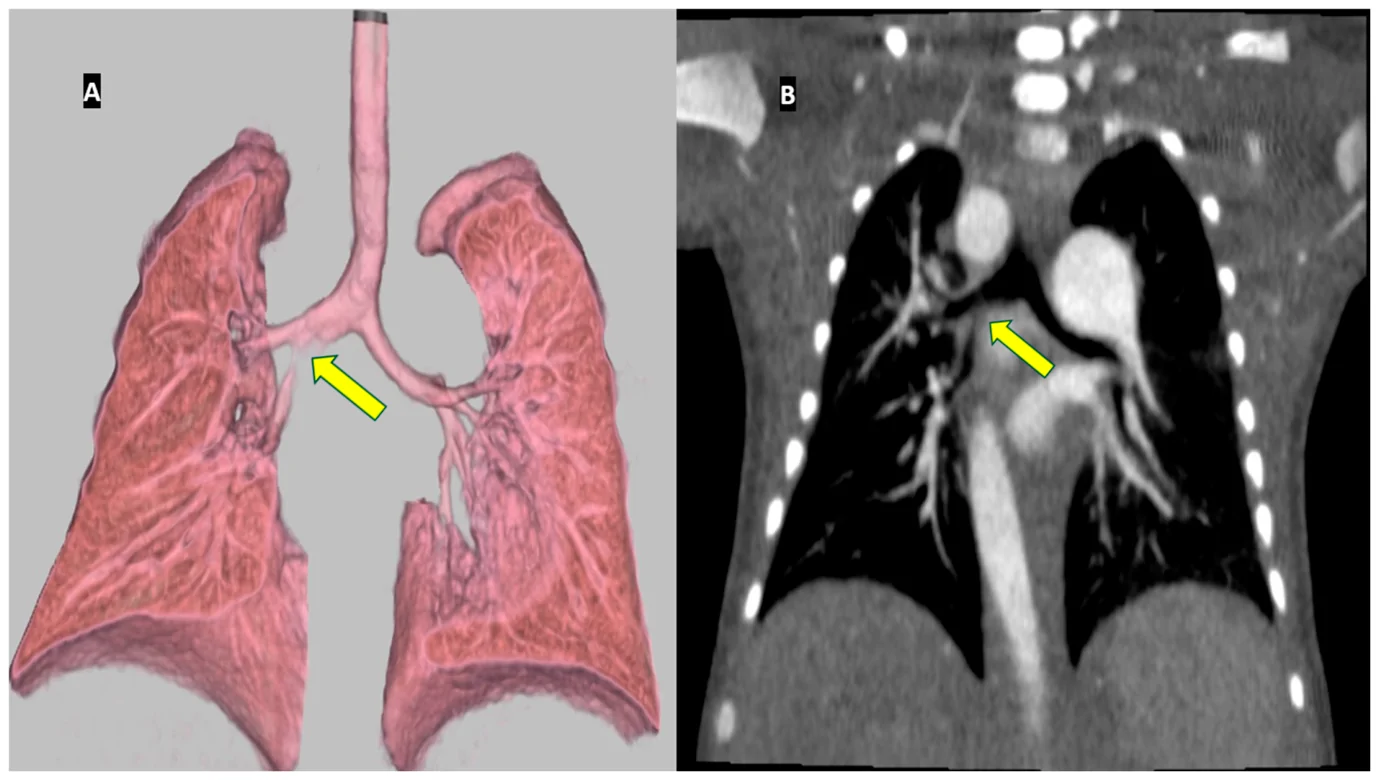
Tracheobronchial compression by the dilated right pulmonary artery in a patient with tetralogy of Fallot with absent pulmonary valve syndrome (TOF-APVS), a hallmark feature of this rare congenital anomaly. (**A**) Three-dimensional CT reconstruction (volume-rendered image) of the tracheobronchial tree displayed alongside the pulmonary parenchyma, demonstrating extrinsic compression of the right main bronchus by the aneurysmally dilated right pulmonary artery (arrow). (**B**) Contrast-enhanced coronal CT image showing the spatial relationship between the dilated right pulmonary artery and the right main bronchus, with severe luminal narrowing extending to the level of the bronchus intermedius (arrow).

Among the 20 patients who underwent CT, airway compression was identified in 13 (65.0%) and was absent in 7 (35.0%) (Figure 2). The location of compression involved the trachea together with both the right and left main bronchi in 5 patients, the right main bronchus alone in 5 patients, the trachea alone in 1 patient, and the left main bronchus alone in 2 patients. Bronchial narrowing was severe in 2 patients and moderate in 11 patients (Table 3).

Pulmonary artery reconstruction/reduction was performed more frequently in patients with airway compression (84.6%, 95% CI 54.6–98.1%) than in those without (57.1%, 95% CI 18.4–90.1%), although this difference did not reach statistical significance (*p* = 0.290).

The rate of at least one reintervention was higher in patients with airway compression (53.8%, 95% CI 25.1–80.8%) than in those without (28.6%, 95% CI 3.7–71.0%), but the difference was not statistically significant (*p* = 0.374). Risk differences with 95% CI for these comparisons are presented in Table 4. None of the differences reached statistical significance in this exploratory subgroup analysis.

Early reoperation (*p* = 0.521) and early postoperative mortality (*p* = 1.000) occurred only among patients with airway compression; however, these comparisons did not reach statistical significance, and the small number of patients limits interpretation. The rate of PPVI was similar between the two groups (*p* = 1.000) (Table 4).

Because CT was not performed in all patients, findings based on CT-detected airway compression should be interpreted with caution.

### 3.3. Preoperative Mechanical Ventilation Requirement (MV+: Present/MV−: Absent)

Preoperative mechanical ventilation was required in 5 patients (18.5%). Early postoperative mortality was more frequent among patients requiring preoperative MV (2/5; 40.0%, 95% CI 5.3–85.3%) than among those not requiring it (0/22; 0.0%, 95% CI 0.0–15.4%; *p* = 0.028). The risk difference for early postoperative mortality was 40.0% (95% CI 8.1–76.9%), representing the only comparison in the subgroup analyses in which the CI excluded zero. Similarly, the length of ICU stay was significantly longer in this subgroup (median 15 vs. 4 days; *p* = 0.008). Duration of intubation (median 7 vs. 2 days; *p* = 0.055) and length of hospital stay (median 17 vs. 10 days; *p* = 0.072) tended to be longer in patients requiring preoperative MV but did not reach statistical significance. The rate of at least one reintervention was similar between the two groups (40.0%, 95% CI 5.3–85.3% vs. 31.8%, 95% CI 13.9–54.9%; *p* = 1.000) (Table 3).

In the CT-evaluated subgroup, airway compression was identified in all patients requiring preoperative MV (3/3; 100%, 95% CI 29.2–100.0%), whereas the corresponding rate in patients not requiring MV was lower (10/17; 58.8%, 95% CI 32.9–81.6%); the risk difference of 41.2% (95% CI −18.3 to 64.0%) did not reach statistical significance (*p* = 0.521) (Table 3).

### 3.4. Surgical Details (N = 27)

Surgical reconstruction consisted of a transannular patch in 16 patients (59.3%) and reconstruction with a valved conduit in 11 patients (40.7%). Pulmonary valve replacement was performed in 13 patients (48.1%); a right atrial appendage (RAA) valve was used in 4 (14.8%) and a polytetrafluoroethylene (PTFE) valve in 9 (33.3%), while no bioprosthetic valve was used. Pulmonary artery reduction/reconstruction (PA plication, reduction arterioplasty, and/or reconstruction) was performed in 19 patients (70.4%) (Table 5).

Overall, 9 patients (33.3%) underwent at least one reintervention (early reoperation, late reoperation, or catheter reintervention). Of these, 7 patients underwent catheter reintervention, 2 patients underwent early reoperation, and 2 patients underwent late reoperation; some patients underwent more than one type of reintervention. Among the 7 patients (25.9%) who underwent catheter reintervention, percutaneous pulmonary valve implantation (PPVI) was performed in 4 (14.8%), balloon angioplasty for conduit stenosis in 2 (7.4%), and balloon angioplasty combined with stent placement in 1 (3.7%). Surgical management and early and late outcomes are summarized in Table 5.

### 3.5. Early Postoperative Outcomes

The median duration of postoperative intubation was 2 days (IQR, 1–3 days; range, 1–30 days), and the median length of ICU stay was 4 days (IQR, 3–8 days; range, 2–36 days). The median length of hospital stay (time to discharge) was 11 days (IQR, 8–16 days; range, 6–41 days); analyses of length of hospital stay were based on 25 patients, as two patients died before discharge. Early postoperative mortality (≤30 days or before discharge) was 7.4% (2/27) (Table 5). Prolonged postoperative intubation exceeding 7 days was required in 6 patients (22.2%); reintubation was required in a subset of patients, although exact numbers could not be reliably retrieved from the medical records.

### 3.6. Follow-Up and Late Outcomes

The median follow-up duration was 30 months (IQR, 7–105 months; range, 1–156 months) for the overall cohort, and 70 months (IQR, 15–110 months; range, 4–156 months) among the 22 surviving patients. Vital status was ascertained for all 27 patients; among the 22 surviving patients, 1 (3.7% of the total cohort) was lost to long-term outpatient clinical follow-up after being discharged in stable condition (last documented visit at 4 months postoperatively). All 3 late mortality events were confirmed through institutional death records, ensuring complete mortality ascertainment. Overall mortality during follow-up was 18.5% (5/27). The causes of early postoperative death were respiratory failure (postoperative day 16) and low cardiac output syndrome (postoperative day 30). The exact causes of the three late deaths could not be established, as these patients had been lost to long-term outpatient clinical follow-up and death was ascertained only through institutional death records.

During the follow-up period, 9 patients required reintervention. The median time to reintervention was 42 months (interquartile range [IQR], 3.5–55.8 months; range, 0.5–102 months), with 3 patients requiring early (≤12 months), 4 mid-term (12–60 months), and 2 late (≥60 months) reinterventions. Late reoperation was required in 2 patients (7.4%; 1 conduit replacement for conduit stenosis and 1 pulmonary valve replacement). Catheter-based reintervention was performed in 7 patients (25.9%), consisting of PPVI for free pulmonary regurgitation in 4 (14.8%), balloon angioplasty for conduit stenosis in 2 (7.4%), and balloon angioplasty with stenting for conduit stenosis in 1 (3.7%). (Table 5). Kaplan–Meier analysis of freedom-from-reintervention showed cumulative reintervention-free estimates of 92.6% at 1 year, 81.2% at 2.4 years, and 51.7% at 5 years. The median freedom-from-reintervention was 102 months (95% CI 29.6–174.4 months).

## 4. Discussion

In this 15-year single-center cohort of 27 patients with TOF-APVS, three principal findings emerged. First, preoperative mechanical ventilation was significantly associated with early postoperative mortality (40.0% vs. 0%; *p* = 0.028) and with a prolonged ICU stay (median 15 vs. 4 days; *p* = 0.008), whereas the differences in duration of intubation and hospital stay did not reach statistical significance but showed a similar directional trend. Second, CT-detected airway compression was a frequent anatomical finding (13/20; 65.0%), often involving the right main bronchus and, in some patients, extending to the bronchus intermedius; however, in our cohort this finding could not be statistically linked to early mortality, late outcomes, or the need for reintervention. Third, the overall reintervention rate during follow-up was 33.3%, driven largely by catheter-based procedures including percutaneous pulmonary valve implantation in 14.8% of patients, which reflects the ongoing longitudinal burden of RVOT dysfunction after initial repair. Taken together, these observations suggest that preoperative mechanical ventilation may serve as a practical clinical indicator of disease severity in TOF-APVS, whereas the prognostic implication of CT-detected airway compression appears to be modulated by selective clinical use of imaging and by indication-based treatment decisions, and warrants reassessment in larger cohorts.

TOF-APVS is a rare variant of tetralogy of Fallot marked by aneurysmal dilatation of the main pulmonary artery and its branches, with resulting tracheobronchial compression and associated tracheobronchomalacia [16] (Figure 1 and Figure 2). Historical series reported considerably high mortality rates in symptomatic infants; however, advances in surgical technique and perioperative care have driven a marked decline in early mortality, which is now typically reported around 10–20% and even lower in more recent series [9,18]. Recognizing airway compression and tailoring the surgical approach accordingly—including pulmonary artery reduction/reconstruction and homograft-based central pulmonary artery replacement in appropriate patients—may improve outcomes [7,10]. In our series, airway compression was identified in 65% of patients who underwent CT, a rate that aligns with previous reports [9,18] (Figure 2). Yet, unlike several series in which airway compression has been linked to postoperative respiratory morbidity and clinical course [9,18], CT-detected airway compression in our cohort could not be statistically associated with either early mortality or reintervention.

Several factors may account for this finding. First, the small size of the CT-evaluated subgroup and the limited number of outcome events likely constrained the statistical power. Second, in clinical practice the CT finding is only one component of the surgical decision-making process; the decision to perform PA reduction/reconstruction also takes into account the severity of clinical symptoms and the degree of PA dilatation [10]. In line with this approach, PA reconstruction was carried out more frequently in patients with airway compression in our series; this selective management strategy may have masked any potential association between airway compression and outcomes (indication-based treatment selection). Taken together, the present data do not preclude a prognostic effect of airway compression but indicate that this association warrants reassessment in larger cohorts.

Unlike anatomical findings, the need for preoperative MV directly reflects the patient’s global physiological status. In our cohort, early postoperative mortality was significantly more frequent, and the length of ICU stay was significantly longer, in patients who required preoperative MV. Duration of intubation and length of hospital stay also tended to be longer in this subgroup, although these trends did not reach statistical significance, likely because of the small sample size. Our findings are in keeping with previous reports; in the national registry series of 98 patients reported by Dorobantu et al., 30-day mortality was markedly higher among patients requiring preoperative ventilatory support (16.7% vs. 1.3%; *p* = 0.04) [19]. Along the same lines, Avdikos et al. observed that preoperative ventilator dependency was associated with earlier surgical repair and a higher reintervention burden [10]. Taken together, these observations suggest that preoperative MV may represent a potential clinical marker of disease severity in TOF-APVS; however, given the small sample size and the exploratory nature of our analyses, this association should be considered hypothesis-generating rather than confirmed, and validation in larger cohorts is required. The observation that all patients requiring preoperative MV in the CT-evaluated subgroup had airway compression further raises the possibility that clinical severity may coexist with anatomical compression and, possibly, dynamic airway vulnerability; larger series with standardized airway assessment are needed to confirm this association.

The overall reintervention rate in our series was 33.3%; catheter-based reintervention was performed in 25.9% (n = 7) of patients, and these procedures consisted of percutaneous pulmonary valve implantation (PPVI) in 14.8% (n = 4), balloon angioplasty for conduit stenosis in 7.4% (n = 2), and balloon angioplasty combined with stent placement in 3.7% (n = 1). Reintervention following TOF-APVS repair is an expected component of the natural disease course, and the demand for reintervention is likely to grow as patients reach adulthood. Talwar et al. reported relatively favorable mid-term outcomes, with freedom from reoperation rates of 93% and 89% at 5 and 10 years, while noting that the need for reintervention is expected to rise with increasing age [18]. Along similar lines, Avdikos et al. emphasized that reinterventions are common in this population [10]. PPVI has emerged as an established alternative to surgery in the management of RVOT dysfunction during late adolescence and adulthood, with low complication rates and freedom from reoperation reported at approximately 86% at 30 months [20,21]. In our cohort, 4 patients eventually required PPVI, which underlines the need for long-term follow-up of RVOT function after initial repair. Overall mortality during follow-up was 18.5% (5/27); 2 deaths (7.4%) occurred in the early postoperative period (≤30 days or before discharge), and 3 (11.1%) occurred after hospital discharge during late follow-up. This pattern suggests that long-term morbidity and mortality in TOF-APVS are not confined to the perioperative period and reinforces the importance of regular cardiology follow-up.

Our findings have both clinical and public health implications. Clinically, preoperative mechanical ventilation identifies a high-risk subgroup requiring careful perioperative planning, while CT-based anatomical characterization of airway compression should continue to guide individualized surgical strategy. The considerable reintervention burden observed during follow-up highlights the need for structured, lifelong cardiology follow-up, including a coordinated transition from pediatric to adult care. From a public health perspective, the rarity and clinical complexity of TOF-APVS argue for the centralization of care within tertiary congenital cardiac centers, where surgical expertise, advanced imaging, and interventional resources can be pooled to optimize both perioperative and long-term outcomes. Given the substantial reintervention burden extending into adulthood, structured transition-of-care programs and multicenter registries are essential to ensure continuity of specialized follow-up and to strengthen the evidence base guiding the management of this rare congenital anomaly.

Certain limitations should be acknowledged. The retrospective single-center design and the modest sample size limit the generalizability of our findings. The selective use of CT in symptomatic patients introduces an inherent selection bias, and the small preoperative MV and airway compression subgroups limited the statistical power to detect associations, as reflected in the wide confidence intervals reported in Table 3 and Table 4; non-significant findings should therefore be interpreted as inconclusive rather than as evidence of no association. The wide age range—from the neonatal period through adulthood—reflects the heterogeneity of the population, further increased in one patient by a delayed surgical repair due to loss to follow-up between initial diagnosis and re-presentation. In addition, the 15-year study period spans an era in which surgical techniques and perioperative management may have evolved; although our institutional approach remained largely consistent, subtle temporal effects on outcomes cannot be excluded. Because subgroup comparisons were hypothesis-generating in nature, no formal correction for multiple comparisons was applied, and *p* values should be interpreted as findings requiring confirmation. Additional limitations include the absence of routine genetic testing for 22q11.2 deletion and the lack of systematic data on postoperative tracheobronchomalacia or bronchoscopic evaluation. Notwithstanding these limitations, our study contributes one of the few single-center experiences from our country to the literature on this rare congenital anomaly, with detailed anatomical characterization of airway compression enhancing the interpretability of the subgroup analyses.

## 5. Conclusions

In this 15-year single-center cohort of 27 patients with TOF-APVS, preoperative MV emerged as a clinically meaningful indicator of a more vulnerable subgroup, associated with markedly higher early postoperative mortality and prolonged ICU stay. CT-detected airway compression was a frequent anatomical finding—often involving the right main bronchus with extension to the bronchus intermedius—yet, in our cohort, its independent prognostic value could not be statistically confirmed, likely reflecting selective imaging and indication-based surgical management. From a practical standpoint, the requirement for preoperative mechanical ventilation may be incorporated into perioperative risk assessment as a readily available bedside marker, while detailed anatomical assessment of airway compression should continue to inform individualized surgical planning. Future research should focus on larger, ideally multicenter, prospective studies with standardized airway assessment and long-term follow-up, in order to better define the prognostic role of anatomical airway compression and to refine risk stratification tools for this rare and clinically heterogeneous congenital anomaly.

## Figures and Tables

**Table 1:** Baseline demographic and preoperative characteristics of patients with tetralogy of Fallot with absent pulmonary valve syndrome (N = 27).

Variable	Value
**Demographic characteristics**	
Sex, n (%)	
Male	17 (63.0)
Female	10 (37.0)
Age at diagnosis (months), median (IQR) [range]	3 (1–8) [0–360]
Age at operation (months), median (IQR) [range]	8 (4.5–16) [0–432]
Weight at presentation (kg), median (IQR) [range]	4.8 (4.0–6.0) [2–55]
**Preoperative status**	
Preoperative mechanical ventilation, n (%)	5 (18.5)
**Preoperative imaging**	
Cardiac catheterization/angiography, n (%)	13 (48.1)
CT, n (%)	20 (74.1)
Airway compression on CT, n/N (%) ^1^	13/20 (65.0)

Data are presented as n (%) unless otherwise specified. ^1^ Airway compression was assessed only in patients who underwent CT (n = 20). Abbreviations: IQR, interquartile range; CT, computed tomography.

**Table 2:** Anatomical and echocardiographic findings of patients with tetralogy of Fallot with absent pulmonary valve syndrome (N = 27).

Variable	Value
**Echocardiographic pulmonary artery measurements**	
Pulmonary valve annulus diameter (mm), median (IQR) [range]	7 (6–8) [4–17]
Pulmonary valve annulus Z-score, median (IQR) [range]	−2.24 (−3 to 0) [−8 to 4]
Main pulmonary artery (MPA) diameter (mm), median (IQR) [range]	16 (12–21) [6–44]
MPA Z-score, median (IQR) [range]	2.9 (2–5) [−4 to 7]
Right pulmonary artery (RPA) diameter (mm), median (IQR) [range]	14.5 (10–20) [6–32]
RPA Z-score, median (IQR) [range]	5.58 (4–7) [1.4–13]
Left pulmonary artery (LPA) diameter (mm), median (IQR) [range]	13 (9–17) [5–30]
LPA Z-score, median (IQR) [range]	5.5 (4–7) [1–10]
**Associated cardiac anomalies**	
Ventricular septal defect (VSD), n (%)	27 (100)
Atrial septal defect (ASD), n (%)	17 (63.0)
Patent ductus arteriosus (PDA), n (%)	3 (11.1)
Aortic arch sidedness, n (%)	
Left aortic arch	18 (66.7)
Right aortic arch	9 (33.3)
**Right ventricular findings**	
Right ventricular dilatation, n (%)	15 (55.6)
Right ventricular dysfunction, n (%)	0 (0)
**Tricuspid valve regurgitation, n (%)**	
None	22 (81.5)
Mild	5 (18.5)

Data are presented as n (%) or median (IQR) [range] unless otherwise specified. Z-scores were calculated according to body surface area–adjusted normative data reported by Pettersen et al. Abbreviations: ASD, atrial septal defect; IQR, interquartile range; LPA, left pulmonary artery; MPA, main pulmonary artery; PDA, patent ductus arteriosus; RPA, right pulmonary artery; VSD, ventricular septal defect.

**Table 3:** Clinical course, interventions, and outcomes according to preoperative mechanical ventilation requirement (N = 27).

Variable	No Preoperative MV (n = 22)	Preoperative MV (n = 5)	Risk Difference (95% CI) ^7^	*p* Value ^1^
**Mortality**				
Early postoperative death ^2^, n (%)	0 (0.0)	2 (40.0)	**40.0 (8.1 to 76.9)**	**0.028**
95% CI	0.0 to 15.4	5.3 to 85.3	—	—
**Reinterventions**				
Any reintervention ^3^, n (%)	7 (31.8)	2 (40.0)	8.2 (−26.9 to 48.2)	1.000
95% CI	13.9 to 54.9	5.3 to 85.3	—	—
**Postoperative course**				
Duration of intubation (days), median (IQR) [range]	2 (1–2) [1–28]	7 (2–23) [1–30]	—	0.055
ICU length of stay (days), median (IQR) [range]	4 (3–5) [2–36]	15 (6.5–23) [5–30]	—	**0.008**
Time to discharge (days), median (IQR) [range] ^4^	10 (8–13.5) [6–41]	17 [range, 15–20] ^5^	—	0.072
**Imaging findings (CT subgroup, n = 20)**				
Airway compression on CT, n/N (%) ^6^	10/17 (58.8)	3/3 (100.0)	41.2 (−18.3 to 64.0)	0.521
95% CI	32.9 to 81.6	29.2 to 100.0	—	—

Footnotes: ^1^ Fisher’s exact test (two-sided) for categorical variables; Mann–Whitney U test (Exact Sig., two-sided) for continuous variables. Bold *p* values indicate statistical significance (*p* < 0.05). Given the exploratory nature of subgroup comparisons and the small sample size, no formal correction for multiple testing was applied. ^2^ Death within 30 days after surgery or before hospital discharge. ^3^ Any reintervention is a composite endpoint defined as the occurrence of at least one of the following events during follow-up: early reoperation, late reoperation, or catheter reintervention. ^4^ Time to discharge analysis included only patients who were discharged alive (n = 25); two patients who died before discharge were excluded from this analysis (n = 22 in the No preoperative MV group, n = 3 in the Preoperative MV group). ^5^ Interquartile range was not calculated for the Preoperative MV group in time-to-discharge analysis because of the limited number of patients (n = 3); only median and range are reported. ^6^ Airway compression was assessed only in patients who underwent CT (n = 20: 17 in the No preoperative MV group, 3 in the Preoperative MV group). ^7^ Individual group proportions with 95% Clopper–Pearson exact confidence intervals (CI) shown in indented rows. Risk Difference calculated using the Newcombe method. Abbreviations: CT, computed tomography; ICU, intensive care unit; IQR, interquartile range; MV, mechanical ventilation.

**Table 4:** Clinical course, interventions, and outcomes according to airway compression on CT in the CT subgroup (n = 20).

Variable	No Airway Compression (n = 7)	Airway Compression Present (n = 13)	Risk Difference (95% CI) ^4^	*p* Value ^1^
Preoperative status				
Preoperative mechanical ventilation, n (%)	0 (0.0)	3 (23.1)	23.1 (−15.3 to 50.3)	0.521
95% CI	0.0 to 41.0	5.0 to 53.8	—	—
**Surgical management**				
Pulmonary artery reconstruction/reduction, n (%)	4 (57.1)	11 (84.6)	27.5 (−10.6 to 61.4)	0.290
95% CI	18.4 to 90.1	54.6 to 98.1	—	—
**Reinterventions**				
Any reintervention ^2^, n (%)	2 (28.6)	7 (53.8)	25.3 (−18.0 to 55.9)	0.374
95% CI	3.7 to 71.0	25.1 to 80.8	—	—
Early reoperation (≤1 year), n (%)	0 (0.0)	2 (15.4)	15.4 (−21.7 to 42.3)	0.521
95% CI	0.0 to 41.0	1.9 to 45.5	—	—
Late reoperation (>1 year), n (%)	1 (14.3)	1 (7.7)	−6.6 (−44.2 to 21.6)	1.000
95% CI	0.4 to 57.9	0.2 to 36.0	—	—
Catheter reintervention, n (%)	2 (28.6)	5 (38.5)	9.9 (−31.3 to 42.9)	1.000
95% CI	3.7 to 71.0	13.9 to 68.4	—	—
PPVI performed, n (%)	1 (14.3)	3 (23.1)	8.8 (−31.1 to 38.4)	1.000
95% CI	0.4 to 57.9	5.0 to 53.8	—	—
**Mortality**				
Early postoperative death ^3^, n (%)	0 (0.0)	1 (7.7)	7.7 (−28.3 to 33.3)	1.000
95% CI	0.0 to 41.0	0.2 to 36.0	—	—

Footnotes: ^1^ Fisher’s exact test (Exact Sig., two-sided). Given the exploratory nature of subgroup comparisons and the small sample size, no formal correction for multiple testing was applied; therefore, *p* values should be interpreted as descriptive rather than confirmatory. ^2^ Any reintervention is a composite endpoint defined as the occurrence of at least one of the following events during follow-up: early reoperation, late reoperation, or catheter reintervention. Component categories are not mutually exclusive; a patient may contribute to more than one component. ^3^ Death within 30 days after surgery or before hospital discharge. ^4^ Individual group proportions with 95% Clopper–Pearson exact confidence intervals (CI) shown in indented rows. Risk Difference calculated using the Newcombe method. Abbreviations: CT, computed tomography; PPVI, percutaneous pulmonary valve implantation.

**Table 5:** Surgical management and clinical outcomes of patients with tetralogy of Fallot with absent pulmonary valve syndrome (N = 27).

Variable	Value
**Surgical reconstruction approach**	
Transannular patch repair, n (%)	16 (59.3)
Valved conduit repair, n (%)	11 (40.7)
**Pulmonary valve replacement**	
Pulmonary valve replacement performed, n (%)	13 (48.1)
Type of valve used	
Right atrial appendage (RAA) valve	4 (14.8)
PTFE valve	9 (33.3)
Bioprosthetic valve	0 (0.0)
**Pulmonary artery management**	
PA reconstruction/reduction, n (%)	19 (70.4)
**Early postoperative outcomes**	
Duration of intubation (days), median (IQR) [range]	2 (1–3) [1–30]
ICU length of stay (days), median (IQR) [range]	4 (3–8) [2–36]
Hospital length of stay (days), median (IQR) [range] ^1^	11 (8–16) [6–41]
Early postoperative death ^2^, n (%)	2 (7.4)
**Late outcomes during follow-up**	
Follow-up duration (months), median (IQR) [range]	30 (7–105) [1–156]
Any reintervention (composite) ^4^, n (%)	9 (33.3)
Early reoperation (≤1 year), n (%)	2 (7.4)
Late mortality ^3^, n (%)	3 (11.1)
Overall mortality, n (%)	5 (18.5)
Late reoperation (>1 year), n (%)	2 (7.4)
Catheter reintervention, n (%)	7 (25.9)
Percutaneous pulmonary valve implantation (PPVI), n (%)	4 (14.8)

Data are presented as n (%) or median (IQR) [range] unless otherwise specified. ^1^ Hospital length of stay analysis included only patients who were discharged alive (n = 25); two patients who died before discharge were excluded. ^2^ Early postoperative death was defined as death within 30 days after surgery or before hospital discharge. ^3^ Late mortality refers to deaths occurring after hospital discharge during follow-up. ^4^ Any reintervention is a composite endpoint defined as the occurrence of at least one of the following events during follow-up: early reoperation, late reoperation, or catheter reintervention. Component categories are not mutually exclusive; a patient may have undergone more than one type of reintervention. Abbreviations: ICU, intensive care unit; IQR, interquartile range; PA, pulmonary artery; PPVI, percutaneous pulmonary valve implantation; PTFE, polytetrafluoroethylene; RAA, right atrial appendage.

## Data Availability

The data presented in this study are available on reasonable request from the corresponding author. The data are not publicly available due to privacy and ethical restrictions concerning patient medical records.
